# Evaluating the cost-effectiveness of maternal pertussis immunization in low- and middle-income countries: A review of lessons learnt

**DOI:** 10.1016/j.vaccine.2020.10.054

**Published:** 2021-01-03

**Authors:** Louise B. Russell, Ajoke Sobanjo-ter Meulen, Cristiana M. Toscano

**Affiliations:** a University of Pennsylvania, Department of Medical Ethics and Health Policy, 423 Guardian Drive, c/o Lauren Counterman, Philadelphia, PA 19104, USA; b Bill & Melinda Gates Foundation, 500 Fifth Avenue North, Seattle, WA 98109, USA; c Institute of Tropical Pathology and Public Health, Federal University of Goiás, Goiânia, Goiás, Brazil. (Instituto de Patologia Tropical e Saúde Pública,Universidade Federal de Goiás, Rua 235, S/N - Setor Universitário, Goiânia-GO CEP 74605-050 Brazil

**Keywords:** Cost-effectiveness, Maternal immunization, Pertussis, Dynamic transmission model, Infant vaccination, Vaccine policy

## Abstract

This issue of *Vaccine* is devoted to papers from a research project that developed two types of simulation models, static and dynamic transmission, to evaluate the cost-effectiveness of maternal immunization to prevent pertussis in infants in low- and middle-income countries (LMICs). The research was conducted by a multinational team of investigators and funded by the Bill & Melinda Gates Foundation to gain an understanding of when and where maternal immunization might be a good public health investment for LMICs. Here we review the project’s central lessons for vaccine policy and research. Models require a lot of data. As most LMICs lack good data, the models were built using pertussis disease burden data from Brazil, a middle-income country with three long-established, independent information systems (disease surveillance, hospitalization, and mortality), on the hypothesis that the disease process is similar across countries. Values for key parameters, particularly infant mortality, infant vaccine coverage, and costs of vaccination and treatment, were then varied to represent other LMICs. The results show that coverage levels of infant whole cell pertussis (wP) vaccine are key to the cost-effectiveness of maternal pertussis immunization. In settings where infant wP coverage is below the threshold thought necessary to eliminate pertussis in the population, 90–95%, maternal immunization is cost-effective, even cost-saving. By contrast, it is very expensive in countries capable of maintaining infant vaccination in or above the threshold range. The research also suggests that, while static models may serve to explore an intervention’s cost-effectiveness initially, dynamic transmission models are essential for more accurate estimates. These findings can help guide policies toward maternal pertussis immunization, but also show that developing better data on neonatal pertussis mortality burden and infant vaccine coverage in LMICs, and on the duration of immunity of currently available pertussis vaccines, are key priorities to support better vaccine policy.

*Bordetella pertussis*, the bacterium that causes whooping cough, is a highly contagious respiratory pathogen. The introduction in the 1950 s-1970 s of infant whole cell pertussis (wP) vaccines in low- and middle-income countries through national Expanded Programs on Immunization (EPIs) led to drastic declines in the incidence of the disease. Recently, however, some countries have experienced resurgences of pertussis, and an increase in pertussis-related deaths among infants, beyond anything expected from the cyclical nature of the disease [1].

Routine immunization of pregnant women with acellular pertussis (ap) vaccine, which confers immunity on infants through transplacental antibody transfer and reduces infants’ exposure to the pathogen by protecting their mothers, has been shown to effectively control pertussis-associated neonatal mortality [2]. To date, many high-income countries have introduced maternal ap immunization, but few low- and middle-income countries (LMICs) have done so. Assessing the value of single-dose maternal ap immunization during pregnancy to prevent disease and death in infants in LMICs requires reliable data on pertussis-associated neonatal mortality as well as a low-cost ap vaccine [3].

Maternal immunization is one of several innovative approaches to reduce neonatal mortality in LMICs [4]. While maternal and infant mortality has been cut in half by initiatives implemented to achieve the millennium development goals, the reduction of neonatal mortality has lagged with approximately 2.5 million newborns dying in 2017 [5]. LMICs’ existing maternal immunization programs, which already provide tetanus toxoid, offer a platform with untapped potential to provide additional maternal vaccines and new maternal vaccines are in development, with those against respiratory syncytial virus (RSV) and group B streptococcus (GBS) furthest along the clinical development path [6]. Although, as noted, maternal ap immunization is already routine in high-income countries, and some middle-income countries, a cost-effectiveness evaluation using a static model concluded that the cost of the vaccine may need to be as low as $1.00 per dose, or less, to be a good public health investment in low-income countries [7]. Such a vaccine may be forthcoming: a low-cost recombinant Tdap vaccine has recently demonstrated higher and more sustained immunogenicity in adults than a Tdap comparator and can be manufactured at significantly lower cost than chemically inactivated Tdap vaccine [8].

The papers in this special issue report the results of a project funded by the Bill & Melinda Gates Foundation to evaluate the cost-effectiveness of maternal Tdap vaccine in LMICs. Both static and dynamic transmission simulation models were developed and compared. Realistic dynamic transmission models, which represent the dynamics of infectious disease, require a lot of data on disease burden and the transmission of disease across a population, data that is lacking in many LMICs. To overcome this problem the team built a dynamic transmission model using data from a middle-income country with good data systems, Brazil, on the assumption, tested by the investigators [9], that the disease process is similar across countries. The Brazilian data, collected from its long-established and good quality disease surveillance, hospitalization, and mortality information systems, are described in [10]. Data on key model parameters which are known to vary among countries – infant mortality from all causes, infant pertussis vaccine coverage, and the costs of vaccination and treatment – were then obtained for different countries to create scenarios representing other LMICs. Using these scenarios, the team evaluated the outcomes of maternal immunization in those situations using the dynamic transmission model.

The key findings from the dynamic transmission model (the *Dynamic Model*, for short) are reported by Kim et al. [11]. The *Dynamic Model* shows that maternal ap immunization may be a reasonable investment for LMICs when infant wP vaccination coverage is below the threshold required to eliminate the disease, thought to be 90–95% [12]. It also shows, however, that when infant wP coverage is in and above that range, the range at which herd immunity comes into play and vaccination begins to eliminate the disease, maternal ap immunization becomes very expensive: $1.3 million per disability-adjusted life-year (DALY) averted. The reason is that there is so little pertussis disease still circulating in the population that even infants in the first months of life, before they receive their own vaccinations, are at very low risk and there is little left for maternal immunization to do. Even if the maternal ap vaccine price was lower -- $5/dose instead of the $9.55/dose currently negotiated by international pooled procurement mechanisms, cost/DALY would still be more than $600,000/DALY at these infant wP coverage levels.

Two scenarios were defined for low-income countries, one to illustrate the situation when infant vaccination coverage is low, using data from Nigeria, and one to illustrate the situation when it is very high, using data from Bangladesh. Vaccination and treatment costs were adapted to each scenario using per capita national income. For these scenarios, the *Dynamic Model* again shows the crucial effects of herd immunity, with maternal immunization projected to be cost-saving in countries with low infant vaccination coverage and very expensive per DALY averted in countries with high infant coverage [11].

The *Dynamic Model* was also used to explore the impact of a possible change in pertussis infant vaccination schedules in low-income countries, from the accelerated schedule currently used in most (primary series doses at 6, 10, and 14 weeks) to a simplified schedule administered at 6 weeks, 14 weeks, and 9 months [11]. The analysis showed that the current accelerated schedule saved money and averted more DALYs than the simplified schedule when infant vaccination coverage was low but that there was little difference between the two schedules in health outcomes or costs when infant vaccination coverage was high. These analyses, however, compare the two schedules only in terms of their impact on pertussis. The vaccines used in routine infant vaccination are combined vaccines that protect against several diseases. It is thus important to emphasize that additional assessment of the two schedules is needed, taking all those effects into consideration.

While only dynamic transmission models capture the transmission of the disease through the population, a key consideration in many circumstances, they are more difficult and resource-intensive to build than static models. Russell et al. [13] compare the results of the *Static Model* [7] and the *Dynamic Model* [11] to explore when static models are adequate for good public health decisions and when the extra effort required by dynamic models is worthwhile. As noted, the estimates from the *Dynamic Model* show that maternal immunization could be cost-saving as well as life-saving at low levels of infant vaccination coverage. When infant coverage reaches the threshold range (90–95%), it becomes too expensive to be cost-effective. By contrast, Russell et al. find that the static model fails to pick up the effect of the herd immunity that emerges at high levels of coverage and estimates costs/DALY only modestly higher at coverages in the 90–95% range than at low infant coverage. These results suggest that static models may serve for the initial exploration of an intervention’s cost-effectiveness against infectious disease; the direction of change and the principal drivers of change were the same in both models, even when the magnitude of change was quite different. When, however, an intervention takes place against a background of vaccination in the rest of the population, a dynamic model is crucial to generate accurate estimates of its cost-effectiveness. This caution does not apply in cases, such as that of maternal immunization to protect infants against group B streptococcus (GBS) disease, in which the rest of the population is not vaccinated.

The modeling revealed some important lessons about the data on which vaccine policies are based. Administrative estimates of infant vaccination coverage are used to guide policy in many countries. They are generally acknowledged to be overestimates but are considered good enough for this use. The investigators initially used administrative coverage estimates, which were usually close to 100% in Brazil, to populate the dynamic model. Using these estimates, the *Dynamic Model* projected that pertussis would be eliminated in Brazil within six years. Those projections are consistent with estimates of the threshold range for pertussis vaccination, 90–95% [12], but not with the continuing pertussis outbreaks in Brazil, particularly the 2010–2014 resurgence [10], nor with evidence that vaccine coverage has declined in recent years [14], [15]. For the models, and the scenarios evaluated with the models, the investigators thus turned to household vaccine coverage surveys [16], [17], [18], [19], which are conducted periodically through the Demographic and Health Surveys and as part of other National Health Surveys in several LMICs. These surveys show substantially lower coverage levels when compared to administrative coverage estimates. Additional studies to better understand these trends and the over-estimation resulting from administrative vaccine coverage data are needed to provide guidance to program managers, policy makers, researchers, and modelers.

Better information is also needed on the duration of immunity conferred by the pertussis vaccines currently used in childhood vaccination programs. The research team found that previously published models used values from 2 to 100 years to represent the duration of immunity of wP vaccine, clearly demonstrating the lack of good evidence about this parameter. It is especially important to assess the duration of immunity of currently available wP vaccines because in recent years traditional manufacturers have withdrawn from the market and new emerging-market manufacturers have entered [20], with the result that much of the evidence in the literature, which evaluated older products, is no longer relevant. Current licensure requirements, which require only that new vaccines prove short-term equivalence to existing vaccines, limit the ability to evaluate the duration of wP vaccine immunity and protection adequately. Finally, as some countries that use wP in their routine childhood vaccination programs have already introduced ap maternal immunization, longitudinal studies to assess its impact and added value are important to further inform and guide policy makers in low- and middle-income countries.

In summary, this special issue presents a cost-effectiveness analysis using a dynamic transmission model to evaluate maternal pertussis immunization in low- and middle-income countries (LMICs). The body of evidence presented is valuable to support immunization policy-making at all levels, including country-level decision making, on the use of additional interventions such as maternal immunization to prevent pertussis in young infants. The results reinforce that reaching and sustaining high routine infant vaccination coverage is the most important policy priority for reducing pertussis disease burden in LMICs. Infant coverage is key to the cost-effectiveness and impact of maternal pertussis immunization: when infant coverage is low, maternal immunization is cost-effective, even cost-saving; when infant coverage is high, maternal immunization is not cost-effective. A low-cost maternal ap vaccine may help make maternal immunization a more cost-effective complement to routine infant vaccination at all infant coverage levels in LMICs with significant neonatal pertussis burden. The findings also reinforce the need for more and better-quality data to help guide policy, especially more robust data on neonatal pertussis mortality, on infant vaccination coverage, and on the duration of immunity of currently available pertussis vaccines.

Funding

Bill & Melinda Gates Foundation Grant OPP1124529

Potential conflict of interest

ASTM is employed by the Bill & Melinda Gates Foundation, which funded the research reported in this issue.

Contributors’ Statement

All authors helped draft the paper, reviewed it for critical content, and approved the final version.
